# Nitric Oxide Synthase Dependency in Hydroxyurea Inhibition of Erythroid Progenitor Growth

**DOI:** 10.3390/genes12081145

**Published:** 2021-07-27

**Authors:** Tijana Subotički, Olivera Mitrović Ajtić, Dragoslava Đikić, Juan F. Santibanez, Milica Tošić, Vladan P. Čokić

**Affiliations:** 1 Department of Molecular Oncology, Institute for Medical Research, University of Belgrade, 11129 Belgrade, Serbia; tijana@imi.bg.ac.rs (T.S.); oliveram@imi.bg.ac.rs (O.M.A.); dragoslava@imi.bg.ac.rs (D.Đ.); jfsantibanez@imi.bg.ac.rs (J.F.S.); milica.tosic@imi.bg.ac.rs (M.T.); 2 Centro Integrativo de Biología y Química Aplicada, Universidad Bernardo O’Higgins, Santiago 8370993, Chile

**Keywords:** hydroxyurea, nitric oxide synthase, erythroid progenitors, cell cycle, apoptosis

## Abstract

Hydroxyurea (HU) causes nitric oxide (NO) bioactivation, acting as both a NO donor and a stimulator of NO synthase (NOS). To examine whether HU effects are NO mediated by chemical degradation or enzymatic induction, we studied human and mouse erythroid cells during proliferation, apoptosis, and differentiation. The HU and NO donor demonstrated persisted versus temporary inhibition of erythroid cell growth during differentiation, as observed by γ- and β-globin gene expression. HU decreased the percentage of erythroleukemic K562 cells in the G2/M phase that was reversed by N-nitro l-arginine methyl ester hydrochloride (L-NAME). Besides activation of endothelial NOS, HU significantly increased apoptosis of K562 cells, again demonstrating NOS dependence. Administration of HU to mice significantly inhibited colony-forming unit-erythroid (CFU-E), mediated by NOS. Moreover, burst-forming-units-erythroid (BFU-E) and CFU-E ex vivo growth was inhibited by the administration of nitrate or nitrite to mice. Chronic in vivo NOS inhibition with L-NAME protected the bone marrow cellularity despite HU treatment of mice. NO metabolites and HU reduced the frequency of NOS-positive cells from CFU-E and BFU-E colonies that was reverted by NOS inhibition. HU regulation of the G2/M phase, apoptosis, differentiation, cellularity, and NOS immunoreactive cells was NOS dependent. Inhalation of NO therapy as well as strategies to increase endogenous NO production could replace or enhance HU activity.

## 1. Introduction

**Scheme 1:**
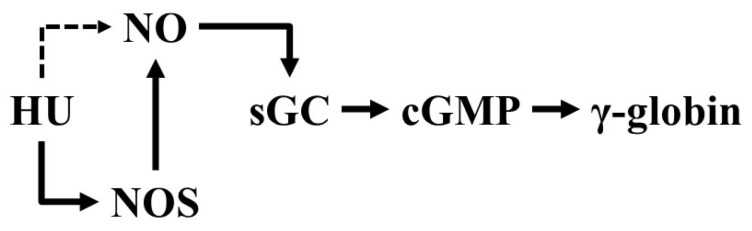
Potentially preferential nitric oxide synthase (NOS) mediated hydroxyurea (HU) induction of γ-globin gene expression via NO/cGMP signaling.

Transient nitric oxide (NO)-like radicals, from hydroxyurea, quench the tyrosyl free radical of the R2 subunit in ribonucleotide reductase [1]. Previous studies have demonstrated that the NO production from hydroxyurea can be mediated by peroxidase and catalase, besides the reaction with hemoglobin to produce iron nitrosyl hemoglobin, nitrite, and nitrate [2,3,4,5]. The NO donors and inducible NO synthase (iNOS)-expressing cells reversibly inhibited DNA synthesis and erythroleukemic K562 cell growth due to the inhibitory interaction of NO with ribonucleotide reductase [6]. NO donors inhibited the growth of colony-forming unit-erythroid (CFU-E) and burst-forming-units-erythroid (BFU-E) growth of CD34+ cells isolated from human bone marrow [7,8,9]. The percentage of S-phase and the total number of BFU-E were inversely correlated with fetal hemoglobin (HbF) levels in the peripheral blood of patients with sickle cell anemia treated with hydroxyurea, which also reduced the total BFU-E colonies [10,11]. In addition, hydroxyurea treatment increased total NO levels and **γ**-globin gene expression in K562 and primary erythroid cells, while NO donors augmented γ-globin and HbF expression in erythroid progenitors [12,13]. Hydroxyurea, in combination with the substrate l-arginine, increased NOS-dependent HbF synthesis in erythroid progenitors [14]. We have demonstrated that hydroxyurea directly interacted with deoxy-heme of soluble guanylyl cyclase (sGC) via the free-radical NO, and activated cGMP production [15] (Scheme 1).

We have shown that hydroxyurea increased endothelial cell production of NO through activation of endothelial NO synthase (eNOS) and posttranscriptional increase in eNOS levels by proteasome inhibition [16,17]. Plasma levels of nitrite and nitrate metabolites of NO (NOx) are significantly increased in patients with sickle cell disease and essential thrombocythemia on hydroxyurea therapy [2,18]. Moreover, NOS activity is increased in patients on hydroxyurea therapy supported by l-arginine [19,20]. Hydroxyurea also enhanced the NOS mediated adenosine triphosphate release from erythrocytes as well as NO production and eNOS levels in endothelial cells [21,22]. 

The NO-mediated hydroxyurea effects on cell differentiation, proliferation, and apoptosis may interpolate to the mechanism of γ-globin gene induction in erythroid cells. Parallel with chemical degradation to NO, we hypothesize that hydroxyurea preferentially stimulates the NOS enzyme to produce NO, which through negative feedback mechanism controls NOS activity. Besides human erythroid cells, we used mice for parallel studies with hydroxyurea and nitrite/nitrate to elucidate NOS enzyme dependency. Separation of enzymatic and chemical generation of NO by hydroxyurea may elucidate its mechanism of action as well as the origin of myelosuppression and HbF synthesis. Harvested mouse bone marrow cells are used for ex vivo expansion of myeloid cultures to examine the effects of NO-producing compounds and the expression of NOS isoforms. Using this approach, we want to distinguish the influence of hydroxyurea induced NO production at the systemic level in the hematopoietic bone marrow microenvironment and in erythroid progenitors.

## 2. Materials and Methods

### 2.1. The Two-Phase Liquid Erythroid Cell Cultures

Peripheral blood mononuclear cells were isolated from buffy coats of normal donors using lymphocyte separation medium (BioWhittaker, Walkersville, MD, USA). We performed a two-phase liquid culture protocol for erythroid differentiation, as previously described [13]. Briefly, after incubation in phase I culture, CD34+ cells were purified by negative selection using the StemSep cell separation method (Stem Cell Technologies, Vancouver, CA, USA). The CD34+ cells were resuspended in the phase II medium, which contained a mixture of cytokines including human recombinant erythropoietin (Amgen, Thousand Oaks, CA, USA). Erythroid cells were treated at different time points in the phase II medium, with S-nitrosocysteine (CysNO) and hydroxyurea (Sigma-Aldrich, St. Louis, MI, USA), incubated at 37 °C in a humidified atmosphere with 5% CO_2_ [13]. The viable cell counts were performed by a trypan-blue exclusion technique (BioWhittaker). For isolation of total RNA from erythroid cells we used the RNeasy Kit (Qiagen, Valencia, CA, USA) according to the manufacturer’s instructions. Quantitative real-time PCR assay of γ- and β-globin mRNA transcripts was carried out with the use of gene-specific double-fluorescently labeled probes in a 7700 Sequence Detector (Applied Biosystems, Foster City, CA, USA) as previously described [13].

### 2.2. Human Erythroleukemic K562 Cell Cultures

Human erythroleukemic K562 cells were cultured in RPMI-1640 medium (Capricorn Scientific, GmbH, Ebsdorfergrund, Germany) with HEPES, 10% fetal bovine serum (FBS, Capricorn Scientific), 100 U/mL penicillin, 100 µg/mL streptomycin, and 2 mmol/L glutamine (Capricorn Scientific) at 37 °C in 5% CO_2_ of relative humidity atmosphere (95%). For eNOS activation, K562 cells were treated up to 30 min. For stimulation of NO production, K562 cells were preincubated 30 min with 0.1 mM L-NG-nitroarginine methyl ester (L-NAME, Sigma Aldrich, St. Louis, MO, USA) and treated with hydroxyurea. For cell cycle, proliferation, and apoptosis analyses, the cells were transferred in 2 mL of RPMI 1640 with HEPES and 2 mmol/L glutamine, 2% FBS, 100 U⁄mL penicillin/streptomycin (Capricorn Scientific) in 6-well plates. Seeded K562 cells were incubated either 24 (1 × 10^6^ cells) or 48 h (0.8 × 10^6^ cells) with NO donors 2-(N,N-diethylamino)-diazenolate-2-oxide, diethylammonium salt (DEANO, t_1/2_ = 2 min), (Z)-1-[2-(2-aminoethyl)-N-(2-ammonioethyl) amino] diazen-1-ium-1, 2-diolate (DETANO, t_1/2_ = 20 h, Alexis Biochemicals, San Diego, CA, USA), and hydroxyurea (Sigma Aldrich) with or without 30 min preincubation of 0.1 mM l-NAME (Sigma-Aldrich). DEANO spontaneously dissociates to liberate 1.5 moles of NO, while DETANO releases 2 moles of NO at 37 °C. The viable cell counts were performed with the use of a trypan-blue exclusion technique (Thermo Fisher Scientific, Waltham, MA, USA).

### 2.3. DNA Cell Cycle Analysis

K562 cell suspensions (0.5 × 10^6^ cells/0.4 mL phosphate buffered saline (PBS) with 2% FBS) were fixed by drop wise addition of ice-cold 96% ethanol and then left on ice for at least 30 min. After centrifugation at 300× *g* for 5 min, the supernatant was carefully aspirated and the cell pellet resuspended. Afterward, the single-cell suspensions were incubated in the water bath with 0.5 mL of RNAase solution (1 mg/mL, RNase A, Thermo Fisher Scientific) for 20 min at 37 °C, and then with 0.5 mL of propidium iodide (PI) (40 µg/mL in PBS w/o FCS, PI, Sigma) for 10 min at room temperature (20 °C ± 2) in the dark. The cellular DNA content was measured using a CyFlow cytometer (Partec GmbH, Münster, Germany). Usually, 3 × 10^4^ cells per sample were analyzed using Flow Max Software (Partec GmbH). Dead cells and debris were gated out based on forward and side scatter. 

### 2.4. Immunoblotting

K562 cells were lysed in chilled RIPA lysis buffer (50 mM Tris-HCl pH 7.6, 150 mM sodium chloride, 1% Triton x-100, 1% sodium deoxycholate, 0.1% sodium dodecyl sulphate, 2 mM EDTA, and 50 mM sodium fluoride) at a ratio of 1 mL of buffer on 1 × 10^8^ cells. A protease inhibitor cocktail (Pierce, Thermo Fisher Scientific) and sodium orthovanadate were added to the lysis buffer just prior to use. Lysates were incubated at 4 °C for 25 min and then centrifuged at 10,000× *g*, 4 °C for 15 min. Protein concentration was determined by the BCA Protein Assay Kit (Pierce, Thermo Fisher Scientific) and the samples were stored at −70 °C until analysis. For western blotting, equal amounts of protein samples were run on polyacrylamide gels and transferred to the polyvinylidene difluoride membrane. The membrane was blocked with 3% milk (Serva Electrophoresis GmbH, Heidelberg, Germany) for 1 h at room temperature (20 °C ± 2) and probed with primary antibodies to β-actin (Abcam, Cambridge, UK), eNOS (Santa Cruz Biotechnologies) and peNOS (Ser1177, R&D Systems, Minneapolis, MN, USA). Peroxidase-conjugated goat antirabbit immunoglobulin (Santa Cruz Biotechnologies) and goat anti-mouse immunoglobulin (Pierce, Thermo Fisher Scientific) were used as secondary antibodies. Hyperfilm was developed to visualize the secondary antibody by the enhanced chemiluminescence reagent system (GE Healthcare, Amersham, UK) according to the manufacturer’s instructions. The content of the examined proteins in cell extracts was estimated by densitometry of the scanned immunoblot band using Image Master Total Lab (GE Healthcare) software. 

### 2.5. Immunocytochemistry 

Mononuclear cells were separated from the bone marrow cells with lymphocyte separation medium (LSM, Capricorn scientific, Ebsdorfergrund, Germany) according to the manufacturer’s instructions. For cytoplasmatic staining, K562 cells, mice bone marrow cells as well as CFU-E and BFU-E/CFU-GM colonies from methylcellulose cultures were collected onto microscope glass slides by cytospins (2 × 10^4^ cells each) and fixed using acetone at room temperature (20 °C ± 2). Samples were treated with 3% H_2_O_2_ solution in PBS to block endogenous peroxidase activity. The next step was incubation with the anti Ki67 antibody (Novocastra Laboratories Ltd., Newcastle, UK), ssDNA (Abcam), iNOS, neuronal NOS (nNOS) and eNOS (Santa Cruz Biotechnologies) antibodies in a humidity chamber overnight in a refrigerator (4 °C). Immunostaining was performed using the streptavidin–biotin technique (LSAB+/HRP Kit, DAKO). Immunoreactivity was visualized with a DAKO Liquid DAB+ Substrate/Chromogen System counterstained with Mayer’s hematoxylin (Merck, Whitehouse Station, NJ, USA) and evaluated under a light microscope. For the negative control samples, normal serum and TBS buffer (1:500) were pipetted without primary antibodies. Proliferative and apoptotic index as well as immunoreactive cells were counted in five and ten high powered fields, respectively, for each sample using a computer-supported imaging system (analysis Pro 3.1) connected to the light microscope (Olympus AX70, Hamburg, Germany) with an objective magnification of ×40. The proliferative and apoptotic rates were expressed as the percentage of KI67- and ssDNA-positive nuclei per total nuclei.

### 2.6. Measurement of Nitric Oxide Synthase Activity

To determine eNOS activity, we used the colorimetric Nitric Oxide Synthase Activity Assay Kit (Abcam, Cambridge, MA, USA) according to the manufacturer’s instructions. For determination of eNOS activity in K562 cells (1.5 × 10^6^), we treated them with hydroxyurea (50, 100, 200 μM) during 1, 6, and 24 h. After treatment, cells were resuspended in NOS Assay buffer at 37 °C. A protease inhibitor cocktail (Pierce, Thermo Fisher Scientific, Waltham, MA, USA), 0.5 M EDTA (Pierce), and sodium orthovanadate (Sigma-Aldrich) were added to the lysis buffer just prior to use. The protein content was assessed with the BCA Kit from Pierce (Thermo Fisher Scientific). In this assay, NO generated by NOS reacts with Griess reagents to produce a colored product with a strong absorbance at OD 540 nm. Applied variation of absorbance in the samples to the standard curve revealed pmoles of nitrite or nitrite activity (pmol/min/μg) generated during the reaction.

### 2.7. Measurement of NO Levels

The Griess method was used to measure the concentrations of nitrite (NO_2_) as an index of the amount of NO in culture medium. The K562 cells (starting 1 × 10^6^ cells) were seeded in RPMI 1640 w/0 Phenol Red medium (Capricorn Scientific) and incubated with hydroxyurea and L-NAME for 6, 24, and 48 h. Plasma was isolated from the mouse blood by centrifugation (2700 rpm, 10 min, 4 °C) to measure nitrite and nitrate levels. It involves the conversion of nitrate into nitrite by vanadium chloride (VaCl_3_). The samples were deproteinized with 0.3 M NaOH and 10% ZnSO_4_ before analysis. The standards were prepared using sodium nitrite or sodium nitrate and deionized water. Each sample was tested in duplicate using 96-well flat-bottomed microplates. For this, 100 µM of each sample and each standard were pipetted into separated wells. Fifty microliters of 2% sulfanilamide (in 5% HCl) was added, followed by 50 µL of 0.1% N-(1 naphthyl) ethylendiamine dihydrochloride (NEDD) in de-ionized water. The plates were incubated at 37 °C for 30 min. After incubation, the absorbance of the reaction mix was read at 540 nm. The mean of duplicate wells for each sample and the standard was taken as the ODs. The nitrite and NOx concentration were determined from linear-regression plots drawn by the ODs of the standards and expressed as µmol/L. These values were subtracted to give the nitrate concentration.

### 2.8. Colony Forming Assays

The experimental protocol was approved by the Ethics Committee of the Institute for Medical Research, University of Belgrade, Serbia, according to the National Law on Animal Welfare consistent with guidelines for animal research and principles of the European Convention for the Protection of Vertebrate Animals Used for Experimental and Other Purposes (Official Daily No. L 358/1–358/6), and the Directive on the protection of animals used for scientific purposes (EU directive 2010/63/EU for animal experiments). Since estrous cycle may modulate the expression of eNOS and iNOS [23], adult male CBA mice (6–8 weeks old, weighing 20–26 g, obtained from the Breeding Facilities of the Military Medical Academy, Belgrade) were used in this study. They were housed six per cage under conventional conditions (lights on at 06:00 h, lights off at 18:00 h, 21 °C) with standard laboratory diet and water provided ad libitum. The animals were treated daily, via the caudal vein, with hydroxyurea (200 mg/kg, Sigma-Aldrich) with or without preincubation of 30 min with NOS nonselective inhibitor L-NAME (50 mg/kg, Sigma-Aldrich) for seven consecutive days according to previous studies [24,25]. In addition, animals were treated with sodium nitrite (1 mmol/kg, Sigma-Aldrich) or sodium nitrate (1 mmol/kg, Sigma-Aldrich). Control mice were treated with PBS. The bone marrow cells from mouse femurs were harvested by aspiration under sterile conditions and monodispersed in Dulbecco’s modified Eagle’s medium (DMEM, Biowest, Nuaillé, France) supplemented with 5% fetal calf serum (FCS, Biowest). Then, 6 × 10^4^/mL bone marrow cells were plated in methylcellulose media (StemCell Technologies, Vancouver, BC, Canada) containing 3 U/mL erythropoietin (EPO, MethoCult M3334) and 3 × 10^4^/mL cells were plated in methylcellulose media containing 3 U/mL EPO supplemented with 50 ng/mL stem cell factor, 10 ng/mL interleukin (IL)-3, and 10 ng/mL IL-6 (MethoCult GF M3434). The cells were plated in duplicate in 24-well tissue culture plates (Sarstedt, Numbrecht, Germany) and incubated at 37 °C in a relative humidity (95%) atmosphere containing 5% CO_2_. Following an incubation period of seven days in MethoCult GF M3434 medium, BFU-E and colony-forming unit-granulocyte/macrophage (CFU-GM) colonies were enumerated using an inverted microscope. CFU-E colonies were scored after three days of culture in MethoCult M3334.

### 2.9. Hematologic Parameters

Whole blood was collected from the control and treated mice, and blood cell counts were made using a hemocytometer. Hemoglobin was analyzed with the cyanmethemoglobin method using Drabkin’s solution (0.1% sodium bicarbonate, 0.005% potassium cyanide, and 0.02% potassium ferricyanide). Values were determined spectrophotometrically at 540 nm and calculated relative to a standard curve. Hematocrit was calculated after brief centrifugation of blood samples in heparinized microcapillary tubes.

### 2.10. Statistical Analysis

One way ANOVA and Dunnett’s post-test were applied using Prism 4 software (GraphPad Software Inc., San Diego, CA, USA). The results were expressed as the mean ± SEM, and differences at *p* < 0.05 were accepted as the level of significance.

## 3. Results

### 3.1. Effects of NO Donors and Hydroxyurea on Erythroid Cell Growth and Proliferation

**Figure 1:**
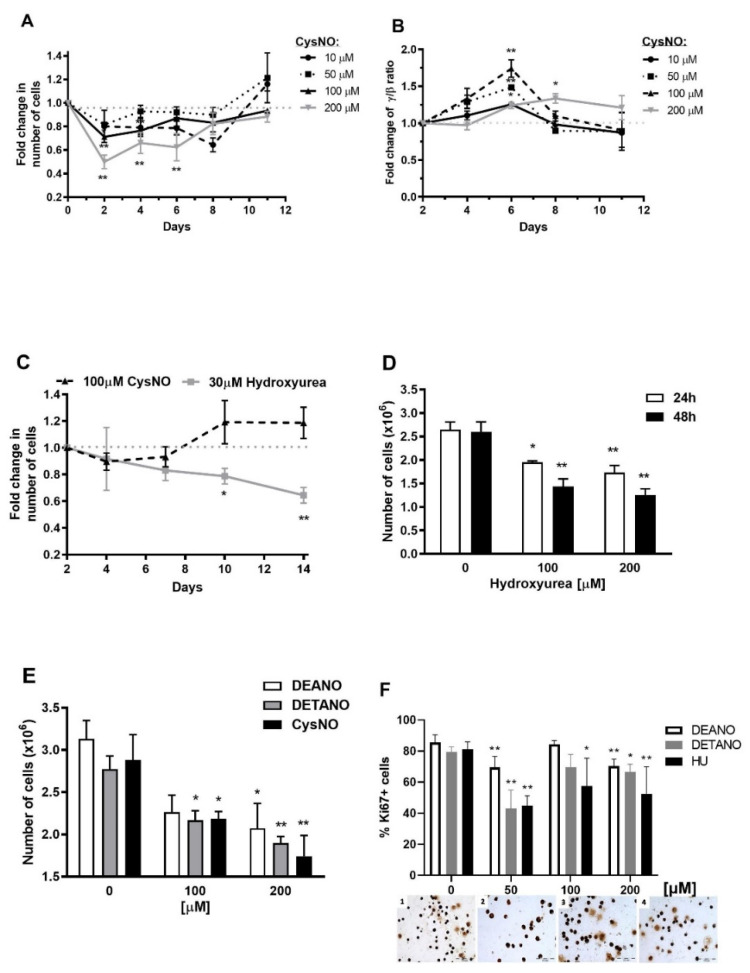
NO donors and hydroxyurea (HU) dose-dependent inhibition of human erythroid and erythroleukemic K562 cell growth and proliferation. Repetitive daily CysNO treatment of human erythroid progenitors and their (**A**) quantification and (**B**) ratio of γ/β globin gene expression at standing days during differentiation in the phase II medium; (**C**) single CysNO and HU treatment (at day 2) of erythroid progenitors; (**D**) 24 and 48 h treatment of K562 cells with HU; (**E**) 24 h treatment of K562 cells with NO donors: DEANO, DETANO, and CysNO; (**F**) Immunocytochemical analyses of the proliferative marker Ki67 positive cells after 24 h of DEANO, DETANO and HU treatment. Immunocytochemistry images correspond to the control and 200 µM of DEANO, DETANO, and HU treatment. We started with 1 × 10^6^ K562 cells. Values are mean ± SEM (*n* = 4–6). * *p* < 0.05 and ** *p* < 0.01 compared to untreated cells.

We selected three NO donors, with various half-life, to expand their comparison with hydroxyurea as an NO releasing or producing compound. We performed a repeated and dose dependent treatment with CysNO during in vitro erythroid differentiation (Figure 1A). The dose-dependent reduction in cell growth was evident during the first six days of erythroid differentiation. In addition, CysNO increased γ/β globin gene expression ratio in human erythroid progenitors (mostly up to day 6) as markers of erythroid differentiation (Figure 1B), similarly to hydroxyurea [13]. Moreover, a single treatment by CysNO at day 2 of erythroid differentiation had temporary inhibition of erythroid cell growth up to day 6 (Figure 1C). In contrast to NO donors, the NO-releasing drug hydroxyurea demonstrated a continuous inhibition of erythroid cells growth, reaching 20–40% after day 6 (Figure 1C). K562 cell growth was reduced with hydroxyurea after 24 h of incubation up to 40%, while prolonged treatment of 48 h showed more prominent inhibition up to 65% (Figure 1D). We demonstrated that NO-releasing agents such as short half-life DEANO and CysNO and long half-life DETANO significantly reduced erythroleukemic K562 cell growth at concentrations of 100 µM and 200 µM after 24 h of incubation of up to 40% (Figure 1E). Immunocytochemical staining for Ki67 demonstrated a high proliferative index for K562 erythroleukemic cells after 24 h (80–85%) of incubation (Figure 1F). Short term NO donor DEANO reduced cell proliferation up to 70%, reaching statistical significance, while DETANO reduced cell proliferation between 42% and 66% (Figure 1F). Hydroxyurea also reduced cell proliferation after 24 h (Figure 1F). Both NO donors and hydroxyurea failed to change K562 cell proliferation rate after prolonged treatment for 48 h. Hydroxyurea and NO overlap in inhibiting the growth and proliferation of erythroid cells, but the sustained effect is attributed only to hydroxyurea.

### 3.2. Hydroxyurea Induction of NOS Activity in Erythroleukemic K562 Cells 

**Figure 2:**
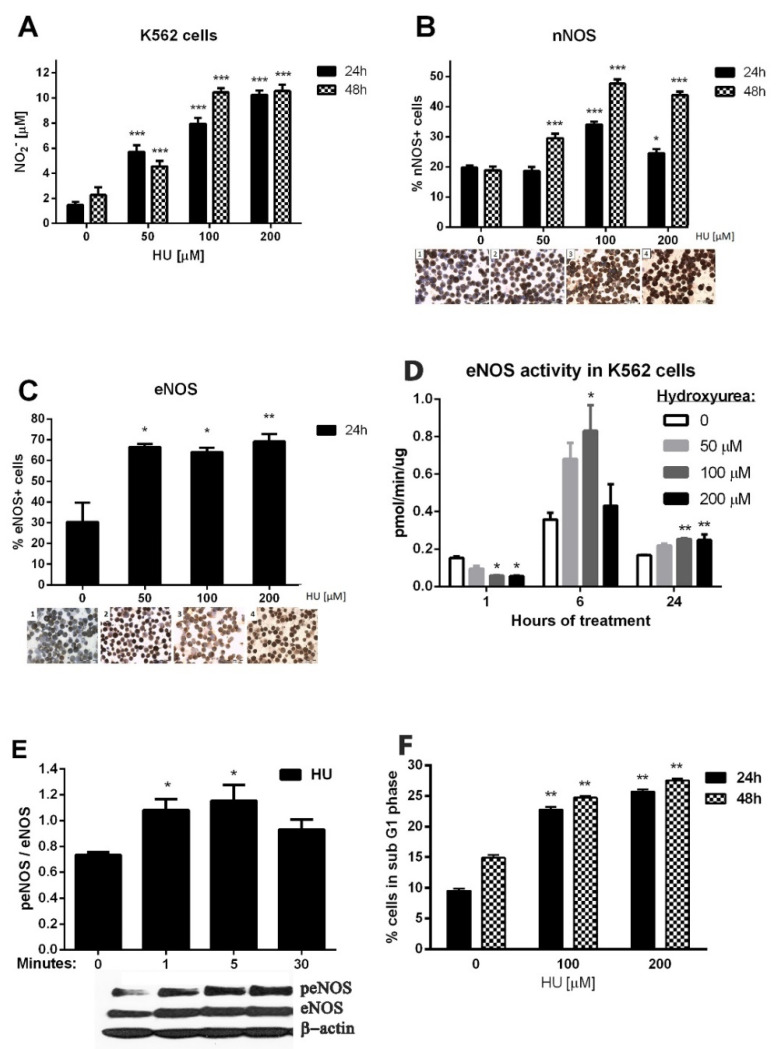
Hydroxyurea (HU) induction of NO in erythroleukemic K562 cells. Level of (**A**) nitrite (NO_2_^−^) and quantity of (**B**) nNOS-positive cells after 24 and 48 h of treatment; (**C**) eNOS-positive cells after 24 h of incubation with HU determined by immunocytochemistry; (**D**) eNOS activity in K562 cells during treatment by HU; (**E**) peNOS/eNOS ratio after 1, 5 and 30 min of stimulation by 100 μM HU; (**F**) Apoptotic cells in the sub G_1_ phase of cell cycle determined by flow cytometry after 24 and 48 h of treatment by HU. Columns correspond to immunocytochemistry (B-after 48 h, (**C**) and immunoblot (**E**) images. Values are mean ± SEM (*n* = 4–5). * *p* < 0.05, ** *p* < 0.01, and *** *p* < 0.001 compared to untreated cells.

Hydroxyurea increased nitrite levels in K562 cells after 24 and 48 h of incubation (Figure 2A). We parallelly analyzed the nitrate and NOx levels during hydroxyurea treatment, but changes did not reach a statistical significance. L-NAME, as a competitive inhibitor of NOS, could not prevent the prolonged stimulation of NO production by hydroxyurea, as measured after 6, 24, and 48 h. We analyzed the presence of NOS isoforms in K562 cells and revealed about 20% nNOS-, 35% eNOS-, and 70% iNOS-positive cells. After prolonged treatment with hydroxyurea, we detected more than double the increase of nNOS- and eNOS-expressing K562 cells (Figure 2B,C). Hydroxyurea could not change already abundant iNOS-positive K562 cells both after 24 and 48 h. Hydroxyurea dose dependently increased eNOS activity in K562 cells after 6 and 24 h of incubation (Figure 2D). Furthermore, hydroxyurea temporary activated the eNOS protein, up to 5 min (Figure 2E). Hydroxyurea stimulated NO production, constitutive NOS levels, and activity in erythroid cells.

### 3.3. Effects of NO Donors and Hydroxyurea on Cell Cycle and Apoptosis

**Figure 3:**
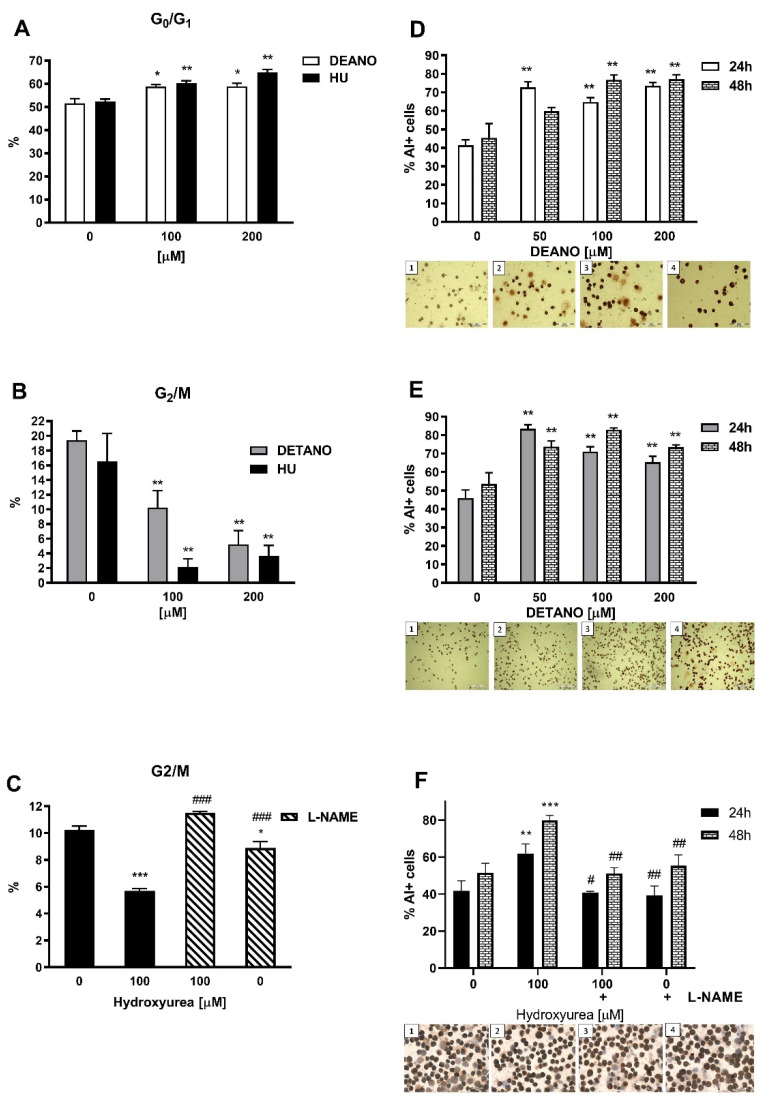
The influence of NO donors and hydroxyurea (HU) on cell cycle and apoptosis in erythroleukemic K562 cells. Using flow cytometry, we measured the percentage of PI positive cells in K562 cells treated with (**A**) HU and DEANO after 48 h at G_0_/G_1_ phase; (**B**) HU and DETANO after 24 h at G_2_/M phase; and (**C**) HU and L-NAME after 48 h at G_2_/M. Apoptotic index (AI) after 24 and 48 h of (**D**) DEANO; (**E**) DETANO; and (**F**) HU treatment with or without L-NAME (100 μM). The apoptotic rate was expressed as the percentage of ssDNA-positive nuclei per total nuclei (apoptotic plus non-apoptotic nuclei). Immunocytochemistry images correspond to results after 48 h. Values are mean ± SEM (*n* = 4–6). * *p* < 0.05, ** *p* < 0.05 and *** *p* < 0.001 compared to the untreated cells (0). # *p* < 0.05, ## *p* < 0.01, and ### *p* < 0.001 compared to only HU treated cells (100).

To examine the mechanism by which NO induces cytostasis and cell cycle, we exposed K562 cells to DEANO, DETANO, and hydroxyurea, and presented their profiles of cell cycle distribution (Supplementary Figure S1). Cell cycle analysis revealed that treatment with DEANO for 48 h resulted in a higher proportion of K562 cells in G_0_/G_1_ (60%) phase of the cell cycle relative to that seen in untreated cells (50%, Figure 3A). After 48 h, the percentage of G_0_/G_1_ phase cells was markedly increased (up to 65%) with hydroxyurea treatment (Figure 3A). After 24 h, 55% and 62% of the cells were significantly arrested in the G_0_/G_1_ phase by 200 μM of DETANO and hydroxyurea, respectively. Furthermore, DETANO and hydroxyurea dose-dependently decreased the percentage of K562 cells in G_2_/M phase after 24 h of incubation (Figure 3B), but not the short-lived DEANO. The percentage of K562 cells in the G_2_/M phase was doubled with the NOS inhibitor L-NAME during hydroxyurea treatment (Figure 3C). This suggests that hydroxyurea inhibition of K562 cell proliferation was NOS dependent. The hydroxyurea stimulation of apoptosis has been shown by apoptotic K562 cells in the sub G1 phase of cell cycle both after 24 and 48 h (Figure 2F). Apoptosis was also detected by DNA fragmentation of increased ssDNA–protein binding interaction using immunocytochemical studies of K562 cells. Short term NO donor DEANO induced apoptosis of K562 cells up to 77% both after 24 and 48 h (Figure 3D). Long term NO donor DETANO also increased apoptosis rate up to 83% in K562 cells after 24 and 48 h (Figure 3E). In addition, hydroxyurea increased apoptosis of K562 cells up to 80% after 48 h, as shown by the apoptotic index (Figure 3F). The hydroxyurea provoked apoptosis of K562 cells has been significantly inhibited by L-NAME both after 24 and 48 h (Figure 3F). Consequently, hydroxyurea stimulation of K562 cell apoptosis was NOS dependent.

### 3.4. Effects of Hydroxyurea and NO Metabolites on Myeloid Progenitor Growth

**Figure 4:**
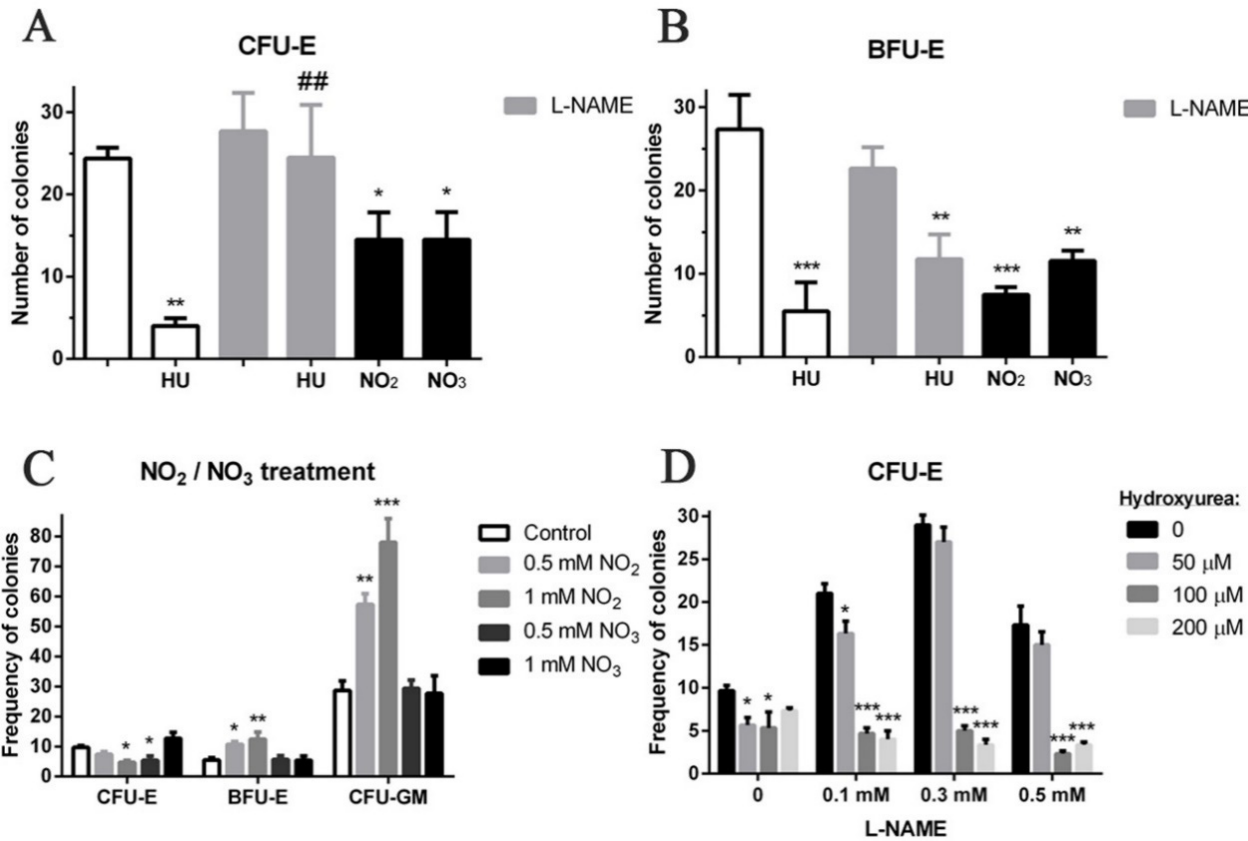
Hydroxyurea and NO derivatives’ induction of erythroid progenitors’ growth of mouse bone marrow origin. Number of ex vivo (**A**) CFU-E and (**B**) BFU-E colonies of mice treated with hydroxyurea (HU, 200 mg/kg), sodium nitrite (NO_2_, 1 mmol/kg), and sodium nitrate (NO_3_, 1 mmol/kg) with or without L-NG-nitroarginine methyl ester (L-NAME, 50 mg/kg) (*n* = 10); (**C**) NO_2_ and NO_3_ induction of mouse CFU-E, BFU-E, and CFU-GM in vitro growth; (**D**) Dose dependent in vitro study of hydroxyurea inhibition of CFU-E growth in the presence of various concentrations of L-NAME (*n* = 3). Values are mean ± SEM. * *p* < 0.05, ** *p* < 0.01, and *** *p* < 0.001 vs. non-treated mice (**A**,**B**) or untreated cells (**C**,**D**); ## *p* < 0.01 vs. HU treated only.

The cytotoxic and NO-dependent effects of hydroxyurea have been examined during mouse myeloid progenitor growth and differentiation. Bone marrow cells harvested from mice, treated in vivo with hydroxyurea for seven days, showed a five-fold reduction in the number of CFU-E colonies (Figure 4A). The obtained myelosuppressive effects of HU were used to observe NOS dependence. This reduction was completely reversed by common NOS inhibitor L-NAME (*p* < 0.01, Figure 4A). The applied concentration of NO derivatives nitrite and nitrate also reduced the quantity of CFU-E and BFU-E colonies (Figure 4A,B). Moreover, hydroxyurea reduced the quantity of immature BFU-E colonies, but L-NAME failed to protect them (Figure 4B). Hydroxyurea (*p* < 0.01) and NO metabolites (*p* < 0.001) inhibited ex vivo CFU-GM colony growth. Hydroxyurea and NO metabolites nitrite and nitrate generally inhibited erythroid colony growth that demonstrated NOS dependence for hydroxyurea in mature colonies. NO metabolites (NO_2_ and NO_3_) reduced in vitro CFU-E colony growth, but nitrite increased the growth of BFU-E and CFU-GM colonies (Figure 4C). Hydroxyurea non dose dependently reduced in vitro CFU-E colony growth, blocked by L-NAME for the lowest concentration of hydroxyurea (Figure 4D). Both ex vivo and in vitro studies confirmed NOS dependence in hydroxyurea reduction of CFU-E colony growth.

### 3.5. Hydroxyurea and NO Effects on Bone Marrow and Peripheral Blood of Mice

**Figure 5:**
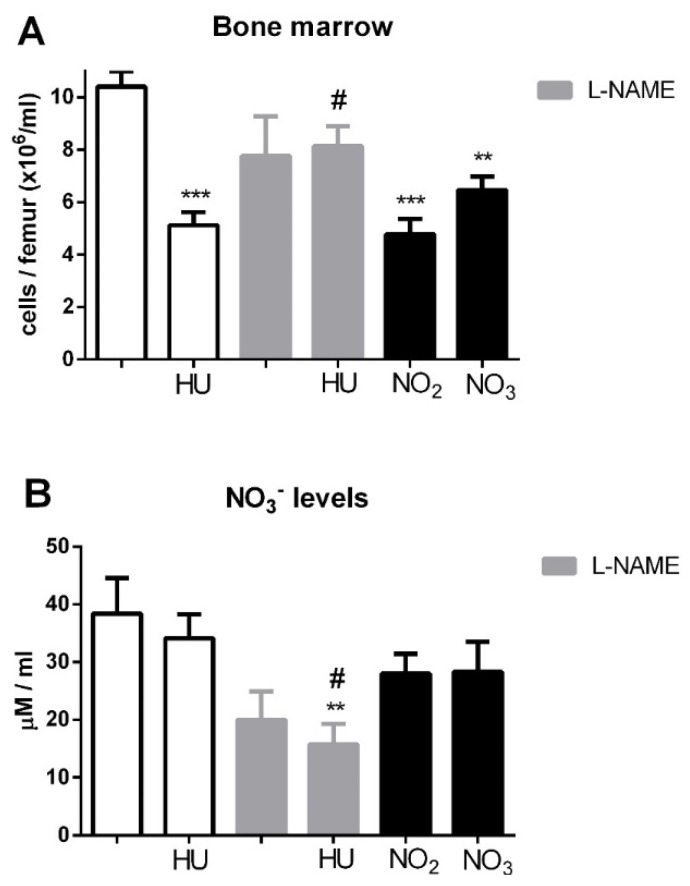
Hydroxyurea and NO derivatives’ induction of mouse bone marrow cells and NO plasma levels. (**A**) Quantity of mouse bone marrow nucleated cells per femur and (**B**) Nitrate (NO_3_-) levels in peripheral blood plasma of mice after seven days of treatment by hydroxyurea (HU, 200 mg/kg), sodium nitrite (NO_2_, 1 mmol/kg), or sodium nitrate (NO_3_, 1 mmol/kg) with or without L-NG-Nitroarginine methyl ester (L-NAME, 50 mg/kg). Values are mean ± SEM (*n* = 10). ** *p* < 0.01 and *** *p* < 0.001 vs. non-treated mice; # *p* < 0.05 vs. HU treated only.

To explore the cytostatic and NO-dependent properties of hydroxyurea in a hematopoietic microenvironment, mouse bone marrow was treated with hydroxyurea for seven days. Hydroxyurea demonstrated a significant decrease in the bone marrow cellularity, which was reversed by L-NAME (*p* < 0.05, Figure 5A). Similarly, both sodium nitrite and sodium nitrate decreased the bone marrow cellularity (Figure 5A). Hydroxyurea significantly reduced the quantity of reticulocytes, leukocytes, and thrombocytes in mice (Table 1). L-NAME prevented the hydroxyurea reduction in reticulocytes (*p* < 0.01, Table 1). Both sodium nitrite and sodium nitrate increased reticulocytes (Table 1). Daily treatment by hydroxyurea did not change NO_3_-level, except in the presence of L-NAME (Figure 5B). In addition, hydroxyurea did not influence NOx level after seven days. Cytostatic effects of hydroxyurea have been confirmed both in bone marrow and peripheral blood, regulated by NOS.

### 3.6. NOS Isoforms Level in Bone Marrow Cells and Erythroid Progenitors after Hydroxyurea and NO Metabolites Treatment

**Figure 6:**
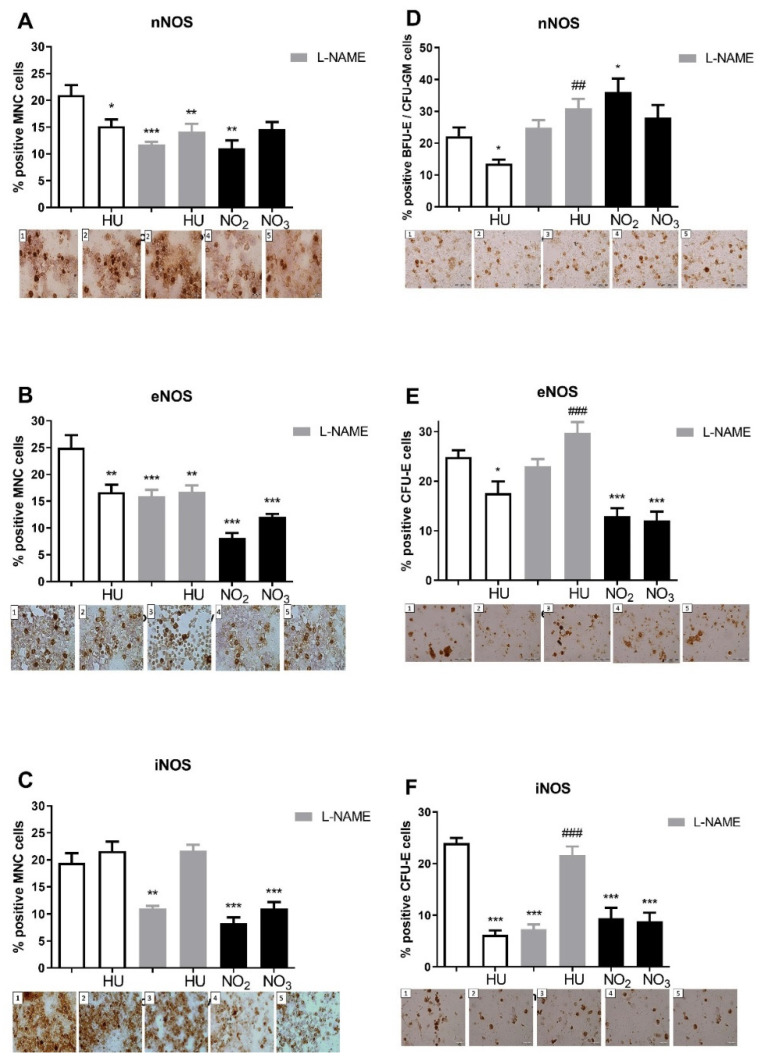
NOS levels in mouse bone marrow derived mononuclear cells (MNC) and erythroid progenitors. Frequency of (**A**) nNOS, (**B**) eNOS, and (**C**) iNOS positive MNC (*n* = 10), (**D**) nNOS positive cells of BFU-E/CFU-GM colonies, (**E**) eNOS, and (**F**) iNOS positive cells of CFU-E colonies (*n* = 5) of mice in vivo treated with hydroxyurea (HU, 200 mg/kg), sodium nitrite (NO_2_, 1 mmol/kg), and sodium nitrate (NO_3_, 1 mmol/kg) with or without L-NG-Nitroarginine methyl ester (L-NAME, 50 mg/kg). Columns correspond to immunocytochemistry images, except for the L-NAME only treatment. Values are mean ± SEM. * *p* < 0.05, ** *p* < 0.01 and *** *p* < 0.001 vs. non-treated mice; ## *p* < 0.01 and ### *p* < 0.001 vs. HU only treated mice.

We analyzed NOS isoforms in bone marrow mononuclear cells (MNC) during prolonged treatment of mice with hydroxyurea and sodium nitrite/nitrate. Frequency of nNOS expressing MNC was significantly reduced by hydroxyurea (*p* < 0.05) and nitrite (*p* < 0.01, Figure 6A). Frequency of eNOS immunoreactive MNC has been reduced by hydroxyurea and nitrite/nitrate as NO delivering agents (Figure 6B). Sodium nitrite/nitrate decreased the quantity of iNOS expressing MNC, although not hydroxyurea (Figure 6C). L-NAME failed to interfere with hydroxyurea induction of NOS isoform levels in MNC (Figure 6A–C). Taken together, hydroxyurea reduced the frequency of constitutive NOS similarly to NO metabolites, except iNOS. NOS levels were further analyzed in ex vivo erythroid colonies of bone marrow origin, derived from mice treated for seven days with hydroxyurea or sodium nitrite/nitrate. The frequency of nNOS positive cells was significantly reduced by hydroxyurea, blocked by L-NAME in BFU-E/CFU-GM colonies (Figure 6D). L-NAME also significantly reversed hydroxyurea’s reduction of eNOS expressing cells in CFU-E colonies (*p* < 0.001, Figure 6E). Furthermore, sodium nitrite/nitrate reduced the frequency of eNOS immunoreactive cells (Figure 6E). Hydroxyurea decreased the quantity of iNOS positive cells in CFU-E, which was prevented by L-NAME (*p* < 0.001, Figure 6F). Similar results were obtained with nitrite/nitrate reduction of iNOS positive cells (*p* < 0.001, Figure 6F). Hydroxyurea demonstrated NOS dependent reduction of NOS immunoreactivity in differentiated erythroid colonies because increased NO levels consequently decreased NOS expressing cells.

## 4. Discussion

In the presented results, NO donors temporarily inhibited erythroid cell growth, in contrast to persistent inhibition by hydroxyurea, while both inhibited proliferation and NOS-dependently reduced the percentage of K562 cells in the G_2_/M phase. Moreover, hydroxyurea increased NO production, constitutive NOS levels, and activity in K562 cells. In accordance, hydroxyurea NOS-dependently increased apoptosis. Hydroxyurea inhibition of mouse CFU-E colony growth, bone marrow MNC, and NOS immunoreactivity of CFU-E/BFU-E cells was also NOS-dependent. Therefore, we have shown a novel NOS dependence in hydroxyurea activities.

Presented hydroxyurea augmentation of eNOS and nNOS expressing K562 cells can be related to the proteasome inhibition, as we already described in endothelial cells [17]. This is consistent with the report that the N-hydroxyurea-based compound reversibly and specifically inhibited a chymotrypsin-like active site of the 20S proteasome [23]. In accordance with our results, it has been shown that the NO donor increased G_0_/G_1_ phase arrest, but decreased the percentage of cells in the early G_2_/M and S phases [26]. In addition, NO donors decreased by half the fraction of cells in the S and G_2_/M phases with a corresponding increase in the G_1_ fraction, suggesting that NO inhibited entry into the S phase, causing cell accumulation in the G_1_ phase [27]. This NO dependent cell cycle arrest was even strongly achieved by hydroxyurea in the presented study. 

In the presented experiments, hydroxyurea and nitrite/nitrate compounds inhibited the ex vivo growth of myeloid colonies derived from mouse bone marrow. Previous human studies have shown that the NO donor inhibits the growth of CFU-E by 30–75%, while the quantity of BFU-E colonies decreased in the presence of hydroxyurea [10,13]. We showed NOS inhibition prevented hydroxyurea reduction of mature CFU-E growth, which supports activation of the NOS enzyme. Hydroxyurea and other known ribonucleotide reductase inhibitors decreased the number of BFU-E colonies and significantly increased HbF, whereas cytotoxic agents (but not ribonucleotide reductase inhibitors) reduced the BFU-E colony number and did not augment HbF levels [28]. Administration of much stronger ribonucleotide reductase inhibitors than hydroxyurea was less effective in reducing the number of mouse bone marrow derived CFU-GM and BFU-E colonies than hydroxyurea [29]. Moreover, two additional ribonucleotide reductase inhibitors with functional moieties similar to hydroxyurea also reduced K562 cell growth and stimulated HbF [30]. Hemoglobin represents the major sink for NO [31], and this variance in HbF levels can be responsible for the observed NOS-dependence only in CFU-E colonies by hydroxyurea. 

The NO-mediated effect of hydroxyurea is of relevance to explain the physiologic role of the NOS/NO/cGMP pathway in globin gene expression. It has been shown that genomic variants rs10901080 and rs10793902 can serve as pharmacogenomic biomarkers for predicting the efficacy of hydroxyurea either by inducing NO biosynthesis or by altering splicing and/or miRNA binding to increase γ-globin levels [32]. The NOS inhibitor L-NAME attenuated the effects of hydroxyurea and L-arginine on HbF synthesis in human erythroid progenitors [27]. Hydroxyurea treatment increased NOx levels and γ-globin transcription in K562 and primary erythroid cells [12]. Furthermore, sGC activators or cGMP analogs increased γ-globin gene expression in K562 cells and primary erythroblasts [33]. In our study, NO metabolites increased hemoglobin levels and significantly reticulocytes in the examined mice. All these reports are in accordance with our previous studies that the NO/sGC/cGMP signaling pathway is responsible for hydroxyurea activation of γ-globin gene expression [13,15]. 

NO and its metabolites nitrite and nitrate are not deposited in tissues up to 48 h after injection, the latter being the main metabolite in urine [34]. This is in accordance with our prolonged daily treatment of mice with hydroxyurea and NO metabolites that did not significantly change their levels in plasma. However, NOS inhibition reduced nitrate levels in plasma. Hydroxyurea and the NO metabolites decreased bone marrow cellularity prevented by NOS inhibition. These observations highlighted the participation of NOS enzyme activation in the hydroxyurea cytotoxic effect. 

The reticulocyte count was significantly decreased in sickle cell disease patients on hydroxyurea therapy [35]. Hydroxyurea also decreased the number of reticulocytes in the presented results, partially prevented by NOS inhibition. In contrast, nitrite/nitrate increased the number of reticulocytes. Hydroxyurea demonstrated a cystostatic effect on leukocytes and thrombocytes. Therefore, the effects of hydroxyurea and NO metabolites were not consistent in hematopoietic mature cells, which are attributable to hemoglobin in peripheral blood [31].

We demonstrated NO metabolites in vivo inhibited the frequency of NOS expressing bone marrow cells, while hydroxyurea preferentially inhibited constitutive isoforms of NOS. During the maturation of erythroid progenitors, eNOS gene expression has been steadily declining as has the production of NO derivatives [15]. The level of eNOS positive granulocytes was reduced by hydroxyurea therapy in patients with myeloproliferative neoplasm [36]. Furthermore, we showed that NO metabolites inhibited the frequency of eNOS and iNOS immunoreactive cells in CFU-E colonies. Patients with sickle cell anemia on hydroxyurea therapy demonstrated decreased iNOS gene expression in neutrophils [37]. This study revealed that hydroxyurea also inhibited the frequency of iNOS expressing cells in CFU-E colonies mediated by NOS. This NOS dependence is analogous to the hydroxyurea inhibition of CFU-E colony growth. The presented results showed that hydroxyurea inhibited the expression of constitutive NOS isoforms in bone marrow cells, but generally reduced NOS expressing cells in erythroid progenitors. This negative feedback regulation of NO producing enzymes by hydroxyurea supports its enzymatic activity.

## 5. Conclusions

This study revealed NOS activation as a mechanism of action by hydroxyurea. NO produced by hydroxyurea and NO metabolites downregulated NOS expression in bone marrow cells and myeloid progenitors. Regarding the mature circulatory cells rich in hemoglobin, hydroxyurea reduced the quantity of reticulocytes with NO dependence. We revealed the NOS mediated hydroxyurea regulation of proliferation and apoptosis, implicating hydroxyurea as a NO prodrug. NO formation and bioactivation are the molecular basis of hydroxyurea pharmacological and anticancer features. Future combination therapies of NO-mediated compounds and ribonucleotide reductase inhibitors may support HbF induction, reduce myelosuppression, and prevent vaso-occlusive crisis in hematologic malignancies and hemoglobinopathies.

## Figures and Tables

**Table 1:** Hematologic parameters in peripheral blood of treated mice.

Treatment	Leukocytes (×10^9^/L)	Erythrocytes (×10^12^/L)	Hemoglobin %	Hematocrit	Reticulocytes	Thrombocytes (×10^5^)
Control	7.6 ± 1.9	6.3 ± 0.7	17.5 ± 2.2	46.3 ± 2.7	67.5 ± 8	10.8 ± 1
HU	6 ± 1.8 *	5.3 ± 1.5	16.5 ± 2.2	44.8 ± 2.1	21.5 ± 1 ***	2.3 ± 0.6 ***
L-NAME	5 ± 0.6 *	4.9 ± 0.2 *	16.4 ± 3.2	48.2 ± 1.1	30.6 ± 7 ***	5.9 ± 2.8 ***
HU + L-NAME	4.8 ± 0.9 ***	5.5 ± 0.3	17.5 ± 1	43.2 ± 3.8	39.5 ± 8 **	3.4 ± 0.6 ***
NO_2_	7.7 ± 1.5	5.6 ± 0.6	19.1 ± 1.4	45.1 ± 1.7	90.5 ± 10 *	9.1 ± 0.6
NO_3_	6.5 ± 1.1	5.5 ± 0.6	18.8 ± 1.4	42.7 ± 3.4	167 ± 23 ***	8 ± 0.3

* *p* < 0.05, ** *p* < 0.01, *** *p* < 0.001 vs. Control (*n* = 10); HU–hydroxyurea; L-NAME–L-N^G^-Nitroarginine methyl ester; NO_2_—Sodium nitrite; NO_3_—Sodium nitrate.

## Data Availability

Data sharing is not applicable to this article.

## Supplementary Materials

The following are available online at https://www.mdpi.com/article/10.3390/genes12081145/s1, Figure S1: Profiles of cell cycle distribution performed on K562 erythroleukemic cells upon propidium iodide staining. Histograms of flow-cytometric analysis show the cell cycle (A) 24 h and (B) 48 h after DEANO treatment; (C) 24 h, and (D) 48 h after DETANO treatment; (E) 24 h and (F) 48 h after hydroxyurea treatment.
