# Supplementary figures and images for: Nitric Oxide Synthase Dependency in Hydroxyurea Inhibition of Erythroid Progenitor Growth
Source: Genes (Basel). 2021 Jul 27;12(8):1145. doi: 10.3390/genes12081145 (PMC8391407; doi:10.3390/genes12081145)

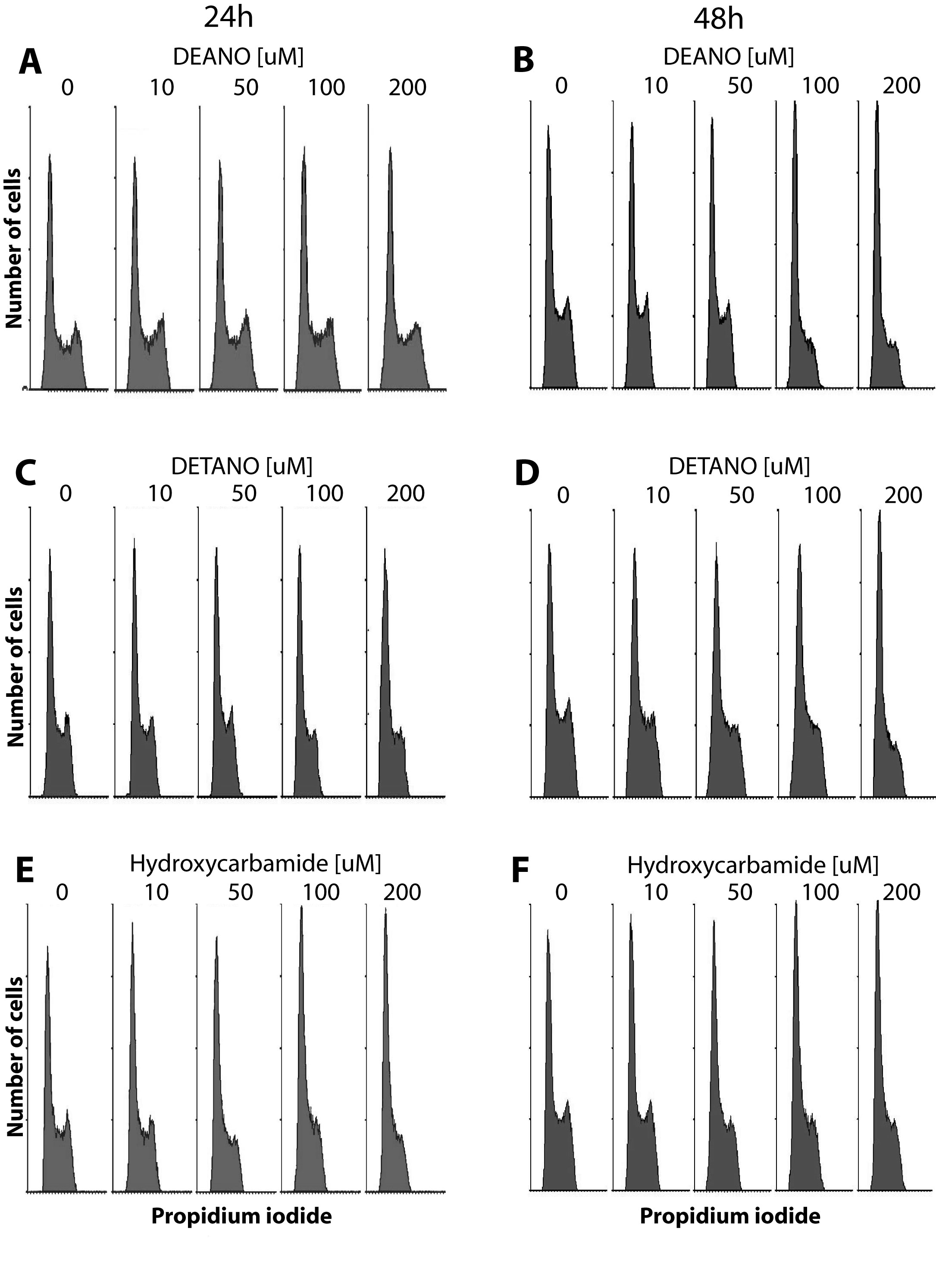

Supplement: Supplementary file 1 [file genes-12-01145-s001.zip › genes-1302502-supplementary.jpg]
